# Prayer Camps and Biomedical Care in Ghana: Is Collaboration in Mental Health Care Possible?

**DOI:** 10.1371/journal.pone.0162305

**Published:** 2016-09-12

**Authors:** Daniel Arias, Lauren Taylor, Angela Ofori-Atta, Elizabeth H. Bradley

**Affiliations:** 1 Yale College, Yale University, New Haven, Connecticut, United States of America; 2 Harvard Divinity School, Cambridge, Massachusetts, United States of America; 3 University of Ghana School of Medicine and Dentistry, Accra, Ghana; 4 Yale School of Public Health, Yale University, New Haven, Connecticut, United States of America; University of Kwazulu-Natal, SOUTH AFRICA

## Abstract

**Background:**

Experts have suggested that intersectoral partnerships between prayer camps and biomedical care providers may be an effective strategy to address the overwhelming shortage of mental health care workers in Africa and other low-income settings. Nevertheless, previous studies have not explored whether the prayer camp and biomedical staff beliefs and practices provide sufficient common ground to enable cooperative relationships. Therefore, we sought to examine the beliefs and practices of prayer camp staff and the perspective of biomedical care providers, with the goal of characterizing interest in—and potential for—intersectoral partnership between prayer camp staff and biomedical care providers.

**Methods:**

We conducted 50 open-ended, semi-structured interviews with prophets and staff at nine Christian prayer camps in Ghana, and with staff within Ghana’s three public psychiatric hospitals. We used the purposive sampling method to recruit participants and the constant comparative method for qualitative data analysis.

**Results:**

Prayer camp staff expressed interest in collaboration with biomedical mental health care providers, particularly if partnerships could provide technical support introducing medications in the prayer camp and address key shortcomings in their infrastructure and hygienic conditions. Nevertheless, challenges for collaboration were apparent as prayer camp staff expressed strong beliefs in a spiritual rather than biomedical explanatory model for mental illness, frequently used fasting and chained restraints in the course of treatment, and endorsed only short-term use of medication to treat mental illness—expressing concerns that long-term medication regimens masked underlying spiritual causes of illness. Biomedical providers were skeptical about the spiritual interpretations of mental illness held by faith healers, and were concerned by the use of chains, fasting, and the lack of adequate living facilities for patients in prayer camps; many, however, expressed interest in engaging with prayer camps to expand access to clinical care for patients residing in the camps.

**Conclusions:**

The findings demonstrate that biomedical care providers are interested in engaging with prayer camps. Key areas where partnerships may best improve conditions for patients at prayer camps include collaborating on creating safe and secure physical spaces and delivering medication for mental illness to patients living in prayer camps. However, while prayer camp staff are willing to engage biomedical knowledge, deeply held beliefs and routine practices of faith and biomedical healers are difficult to reconcile Additional discussion is needed to find the common ground on which the scarce resources for mental health care in Ghana can collaborate most effectively.

## Background

Mental health is an essential part of human wellbeing, yet services and care for patients with mental illnesses are deficient globally [1]. In sub-Saharan Africa, mental health has been particularly under-prioritized [2,3]; in Ghana, for example, only 2% of patients with mental illnesses have access to biomedical treatment [4,5]. As in other countries with large biomedical treatment gaps, a substantial number of people with serious mental illnesses in Ghana rely on traditional and faith healing to meet the need for treatment [6–11]. Many patients and their family seek relief from mental health disorders at prayer camps, predominately Christian facilities run by faith healers where patients seek spiritual healing for their illness [12]; such camps have been documented in Nigeria [13], Togo [14], and Ghana [15].

Given the profound lack of medical resources and the prevalence of the use of faith healing in low-income countries, there is ongoing debate over whether intersectoral partnerships between biomedical care providers and faith healers may be an effective strategy for overcoming the treatment gaps for serious mental illness in such contexts [16]. In Ghana, qualitative exploration of intersectoral partnerships between community mental health units and prayer camps has suggested that partnerships may be viable when founded on mutual respect and bilateral understanding [6]. Divergent perspectives on the underlying cause of and best treatment for mental illness, and concern over human rights violations in prayer camps—including the chaining and forced fasting of patients [12,15,17,18] have been previously identified as potential challenges for faith healer collaborations with biomedical psychiatric services [6]. Less studied has been how prayer camp staff regard intersectoral partnerships, and whether their beliefs and practices provide sufficient common ground with biomedical staff in order to enable cooperative relationships. Addressing this paucity of data could provide critical information needed in assessing the viability of intersectoral partnerships and pathways for addressing gaps in mental health treatment [19].

Accordingly, we sought to examine the beliefs and practices of prayer camp staff, with the goal of characterizing interest in—and potential for—intersectoral partnership between prayer camp staff and biomedical care providers. We also sought to document the perspectives of biomedical care providers vis-à-vis prayer camps and potential intersectoral partnerships. We pursued this goal in Ghana, which provided an ideal setting for this research as the country has recently passed a new Mental Health Act [20] and is seeking ways to expand community-based mental health services [21] and regulate prayer camps [22]. With 96% of the population self-identifying as religious, Ghana has been designated one of the most religious countries in the world by Gallup International [23]. Approximately 71.2% of the population identifies as Christian, while 17.6% of the population identifies as Muslim [24]. Given the prevalence of religious belief within Ghana and limited biomedical resources for mental health—approximately a dozen practicing psychiatrists are available, to serve a population of 25 million people—Ghana is considering whether and how to integrate prayer camps into the formal mental health system [22,25]. The issue is particularly salient amidst international pressure to address issues regarding the treatment and living conditions of patients residing in prayer camps as well as those with mental illness in Ghana generally [17,18,26]. Findings from this study may inform efforts to craft a partnership model that provides for mutual respect, collaboration, and engagement of biomedical care providers and faith healers in mental health care delivery.

## Methods

### Ethics Statement

All study participants provided their verbal informed consent to participate in this study. Based on the research protocol, the Yale University Human Subjects Research Committee determined that verbal, rather than written consent, was appropriate. The study was approved by the Yale University Human Subjects Research Committee. Study protocols and interview guides were additionally reviewed by the medical administrators of each psychiatric hospital visited; the same was made available to prayer camp staff in advance of interviews. No interviews were conducted without the permission of either a hospital administrator or a senior prayer camp staff or leader.

### Setting

Most mental health provider resources in Ghana are concentrated in the Central and Greater Accra Regions, where Ghana’s three public psychiatric hospitals—Accra Psychiatric Hospital, Pantang Hospital, and Ankaful Hospital—are located. These hospitals have a total of 1,322 beds [5]. The number of prayer camps in Ghana is unknown, but has been estimated to number in the hundreds [22]. Camps are commonly thought to vary in size, from small shack-like structures to entire village-communities with large church halls, facilities, and commerce [15]. Most prayer camps are under-resourced, lacking adequate shelters, bedding, mosquito nets, cleaning supplies, and improved sanitation facilities [15,17]. Prayer camps are often staffed by caretakers, pastors, and church elders, and may be led by a prophet or prophetess, who acts as the chief healer, spiritual leader, and administrator of the camp.

### Study design and sample

We conducted a qualitative study using open-ended, semi-structured interviews with prayer camp staff and biomedical health care professionals. We selected a qualitative study because of the limited research on prayer camp practices, resources, and beliefs, and because semi-structured interviews provided participants with the greatest latitude in relating nuanced concepts and practices. Interviews were conducted during June and July 2014 throughout a seven-week period. Nurses and other biomedical staff were recruited from each of Ghana’s three psychiatric hospitals. Known prayer camps were identified by psychiatric hospital administrators, nurses, and members of the Ghana Health Service, as well as a review of the relevant literature [15,18]. Our study included nine prayer camps in the final sample; of the prayer camps identified (n = 10) and approached (n = 10), one declined to participate in the study.

Study participants were purposefully selected to represent a variety of professional roles and geographic locations in order to have a diverse study sample (n = 50 participants across nine prayer camps and three psychiatric hospitals). The prayer camp sample included prophets (n = 4), church elders (n = 5), pastors (n = 2), a reverend, a church member, and a caretaker. The hospital sample included registered mental health nurses (n = 29), general practitioners (n = 2), an orderly, a hospital administrator, a psychiatrist, a community mental health officer, and a community psychiatric nurse interviewed on-site at a prayer camp (which was part of the nurse’s catchment area for community outreach).

The interviewer (DA) provided potential study participants with information regarding the nature of the study, the aims, and the scope of questions that would be asked, prior to requesting consent to participate in the study. The interview guide was provided to participants in advance of obtaining their voluntary oral consent for the interview.

### Data collection

Interviews were conducted in person and on-site in English, Twi, or Fante, based on the request of the participant and with the aid of a translator. Interviews were conducted one-on-one (n = 34), unless the participants preferred that others be present for a group interview (18 participants were interviewed one-on-one and 16 participants were interview in groups, with an average of three participants per group interview). Interviews were audio-recorded with the permission of the participant. Some participants declined permission for audio-recording (n = 5); in these cases, the interviewer instead took notes on interviews by hand, and typed them shortly thereafter. A professional, independent transcription service transcribed the audio-recorded interviews verbatim; transcripts were subsequently reviewed by the interviewer to ensure quality and accuracy. Interviews were conducted using a discussion guide (S1 File), with follow-up prompts to elicit further detail [27,28]. The discussion guide covered five thematic areas: 1) availability of resources (infrastructure and commodities) within the camp; 2) beliefs regarding causes of mental illness; 3) beliefs regarding the effective treatment of mental illness; 4) practices of prayer camps in the treatment of mental illness; and 5) views about collaborations between prayer camps and biomedical mental health care providers.

The interviews lasted between 20 minutes and 1.5 hours, with an average duration of 30 minutes. Additional interviews were conducted until the point of theoretical saturation, defined as the point at which no new concepts emerged through subsequent interviews [29,30].

### Data analysis

The study team conducted qualitative data analysis using the constant comparative method [30,31] as utilized in health services research [32]. First, the lead author inductively developed a preliminary code sheet. Two members of the research team (DA, EB) then independently conducted separate line-by-line coding of 10 transcripts to identify concepts that may constitute codes. Through ongoing discussions, the two readers created a code sheet that reflected the key concepts that emerged from the first 10 transcripts and then applied these to the remaining transcripts. Codes were combined, their meaning refined, and, in some cases, new codes were added until a final code sheet was attained. After developing the final code sheet, three coders (DA, LT, EHB) independently applied codes to the transcripts. Disagreements about coding decisions were resolved by negotiated consensus.

## Results

### Overview of prayer camp settings and resources

**Fig 1:**
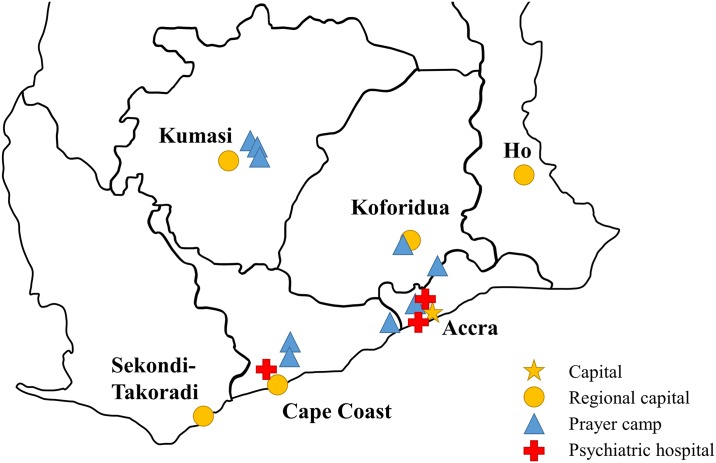
Map of Southern Ghana and location of prayer camps and hospital visited.

The nine prayer camps visited for the study varied in size, age, and geographical location. All of the camps were located in the south of Ghana (Fig 1), generally in rural areas located between 5 and 40 kilometers from the nearest city (e.g., Accra, Cape Coast, Kumasi, or Koforidua). Some of the camps were founded within the last decade; others predated Ghana’s independence in 1957. All of the camps were Christian, and most identified as either Pentecostal or non-denominational.

Of the camps visited, between 3 and 31 church members worked as elders, reverends, and pastors to provide religious services at the camp. The camps hosted church congregations of 100 to 500 members for daily and weekly services. On average, camp staff reported fewer than a dozen patients in residence whom prayer camp staff described as having a serious mental illnesses; staff at one camp, however, stated that they supervised more than 100 patients with serious mental illness. Most patients at prayer camps did not come to the camp with mental illness as their chief concern; more often, physical illness or non-medical problems were primary.

Most of the camps required that relatives care for patients on-site during recovery. These family members acted as caretakers, responsible for the food, clothing, and hygiene of their relatives. None of the prayer camp staff interviewed was able to provide a figure for the average length of stay for patients with mental illnesses; pastors indicated that the length of stay could range from a few days to a year.

Each of the camps was led by a prophet or prophetess. The prophet was the founder of the camp, or had inherited the camp from a relative. The prophet served as the administrative head of the camp and the coordinator of healing. Pastors described the prophet’s responsibilities as assessing the spiritual problems of patients, prescribing treatment regimens (such as the length of fasting), and determining when the patient was fully healed.

Resources and facilities varied widely among the prayer camps in the study. Most camps had a central church, with dormitories and additional structures located nearby. Two of the camps had cars and buses, which were used to ferry congregation members to and from church. Larger camps had security walls, stores, and additional buildings, which served as schools, kitchens, and congregation halls. The largest camp had more than fifty buildings, with its own restaurants, pharmacies, stores, farms, school and police station.

Dormitories for patients with mental illnesses ranged from exposed, outdoor cots covered in bamboo canopies to enclosed dormitories with mosquito nets and bedding. Inadequate structures and measures to protect against rain, prolonged direct sunlight, and mosquitos were found at a majority of the camps visited. Many camps did not have sufficient dormitory space to separate patients with serious mental illness from other residents; staff interviewed indicated that this made living with particularly aggressive patients dangerous for other prayer camp residents.

Staff interviewed at each prayer camp identified financial and material needs that the camp was struggling to address alone. In addition to the challenge of providing care for patients with mental illnesses, prayer camps struggled with providing services to the much larger number of patients who seek treatment for non-mental health related conditions. While some camps charged patients and their relatives for their stays, several of prophets interviewed stated that they either did not charge patients with serious mental illnesses or that they only charged those who were able to pay. To provide services, these prophets noted, prayer camps were reliant on either donations from patients or the support of associated church congregations, in the form of alms and offerings. Prophets also stated that resources were particularly strained when the camp needed to provide care for patients who were abandoned. Abandonment was reportedly due to the difficulties some families had caring for relatives with serious mental illness and the intense stigmatization of people with mental illness. When patients were abandoned, camp staff said that it fell on the camp to care for the patient.

### Causes of mental illness

Prayer camp staff universally reported a belief in spiritual causes of mental illness, which they drew in contrast to the physical aspects of disease. Although some staff recognized that physical issues may contribute to mental illness, the primary cause they uniformly noted was spiritual. As illustration of this belief, one prophetess expressed the following:

*In mental illness*, *98% of the time it is spiritual*. *There is a verse from the Bible*, *Ephesians 6*:*12*. *“For we wrestle not against flesh and blood*, *but against principalities*, *against powers*, *against the rulers of the darkness of this world*, *against spiritual wickedness in high places*.*” That is our philosophy [about] mental illness*.

—Prayer camp prophetess

Demonic possession, whereby a demon entered the host and caused the affliction, was commonly described as at the root of mental illness by prayer camp staff. As illustrated by one pastor, demonic possession was the consequence of leaving oneself vulnerable to the occult.

*The demons can possess a person based on the way you lead your life*. *Sometimes you never accept Christ as your personal savior*. *If you refuse to study the scriptures so that you can be filled with the spirit and covered with the blood*, *then the demons can possess you easily*.

—Prayer camp pastor

Prayer camp staff additionally noted that possession could arise from ancestral curses, particularly if an ancestor had interactions with witchcraft; this, staff elaborated, explained why certain individuals with no personal involvement with the occult could still be possessed as a consequences of ancestry, explaining the observed hereditary pattern of certain cases of mental illness.

*There are a lot of causes*. *Some [patients] are cursed…in the blood…before you trace [the mental illness]*, *you can see that you find it in the family [of the patient]…when you trace it back*, *you see that maybe that person who had it [earlier in the family history] was cursed*.

—Senior prayer camp pastor

With regard to physical causes of mental illness, prayer camp staff considered substance use a common cause of mental illness; however, the staff viewed the impulse to use substances as a manifestation of the demonic spirit’s influence. As one pastor described, this spirit was believed to manifest as the addictive influence of drugs of abuse:

*Some causes are physical. Substance abuse, like cannabis, cocaine, alcohol…It is the spirit that asks them to do it. You tell the spirit to stop, that you will not do this again, but you can't. Unless you add prayer, you don't have the strength to overcome the thing*.

—Pastor

In contrast to prayer camp staff, most biomedical providers emphasized that mental illness arises from physical, not spiritual, factors. Many biomedical providers, however, recognized that their patients often understood and processed their experience of mental illness through a spiritual lens. Among the minority of biomedical providers who indicated that they *personally* believed in spiritual causes of illnesses, informants emphasized that they distinguished between their personal and professional views on mental illness. Some nurses indicated that training to be a mental health nurse instilled a biomedical perspective that replaced personally held spiritual beliefs regarding the source of mental illness. This strongly held view that environmental, biochemical, and somatic factors—rather than spiritual ones—caused mental illness was exemplified by this psychiatric nurse:

*With mental illness, you have issues of environment, head trauma, heredity and genetics, neurochemistry (with the imbalance of dopamine and serotonin), and the abuse of substances. Some infections … can also cause mental illness. People who are not as educated usually think [mental illness] is caused by curses or witches. For me, I don’t believe in [that]*.

—Nurse

The biomedical providers generally recognized that their views differed from those of prayer camp staff, as expressed by a community mental health worker who believed in biomedical causes of mental illness and was critical of faith healers’ approaches to mental illness.

*It’s very difficult to differentiate between psychiatric conditions and when… you are hearing the voice from God*. *When the person really has a psychiatric condition—a known psychiatric condition—and says he’s hearing [a] voice from God*, *and he’s seeing things in dream while he’s fasting*…w*e [the biomedical staff] believe that he’s maybe hallucinating*. *Maybe he’s undergoing the signs and symptoms of [a] psychiatric condition*… *[The faith healers] will believe anything that the patient says*, *any form of hallucination*. *In talking to this person*, *they will believe it*, *that actually there is [a] spirit talking to them*.

—Community psychiatric nurse

### Effective treatments for mental illness

Prayer camp staff reported that the only way for patients with mental illnesses to fully heal was to address the underlying spiritual causes of the illness; until this was accomplished, patients would not be relieved of the physical signs and symptoms of mental illness. This belief was illustrated by the following quotation from a prayer camp pastor.

*First is the spiritual*, *before the physical… The [psychiatric] medicine won’t work [and] the physical issue won’t resolve itself until the spiritual is addressed first… The healing actually comes spiritually before it manifests itself physically*.

—Prayer camp pastor

Some prayer camp staff reported that they encouraged families of patients evidencing symptoms of serious mental illness to first visit a hospital or primary health center in order rule out cases of physical origin, or, in extreme cases, to receive medical sedation; this, however, was not a commonly reported practice among prayer camp staff interviewed in the study.

In contrast, biomedical providers expressed a belief that mental illness required immediate attention. Nurses noted that it was difficult to assess whether mental illnesses were biomedical or spiritual in nature, and that it was therefore necessary to first administer biomedical treatment to “rule out” biomedical conditions. Some nurses said that if prayer camps were unwilling to allow for biomedical treatment to occur before spiritual efforts, perhaps the two could take place concurrently as a compromise.

Biomedical providers who maintained beliefs in the spiritual aspects of mental illness identified hospitals, rather than prayer camps, as the better environment for care, as patients in hospitals could receive both biomedical services in ways that supported their personal religious beliefs. These participants highlighted the availability of religious services in psychiatric hospitals, and the general accommodation of religious beliefs in the course of treatment.

### Practices at prayer camps

Prayer camp staff reported fasting as one of the most common healing practices used. Fasting, as it is generally practiced in the prayer camps, refers to the enforced abstinence from certain or all food and drink for a defined period of time; within Pentecostal theology globally and within many Christian churches in Ghana, fasting is a regular practice among congregants. Staff interviewed expressed the belief that fasting and prayers were the most integral parts of treating mental illnesses, as both were mentioned explicitly by the Bible in Matthew 17:21 as the ways to drive out possessing demons (“But this kind does not come out except by prayer and fasting,” International Standard Version).

While prayer camp staff universally stated that fasting was an important part of treating mental illnesses, staff were divided as to whether patients themselves had to fast, or whether others could fast for them. For some camps, fasting was an expected element of healing. The length of fasting—and the foods prohibited by the fast—varied depending on the particular illness or affliction; a typical case might involve no food or water from 6 AM to 6 PM for three days. Prayer camp staff expressed the belief that fasting, though exhausting for the physical body, provided strength for the spirit. In other camps, fasting was viewed as not necessary for all patients. At these camps, prayer camp staff only encouraged patients without serious mental illnesses to fast, encouraging relatives or pastors to fast in the place of those unable to fast themselves.

All prayer camp staff noted that caning (the beating of patients with canes or sticks) and corporal punishment were practiced at some prayer camps but emphatically rejected it in their own camps. Some described the beating of patients as running counter to a belief in spiritual healing, as it was observed by one pastor that “God did not beat out the demons.” In general, beating a patient was viewed as causing physical harm to the body, without any effect on the spiritual cause underlying the mental illness within it. A quotation from a camp prophetess typified similar statements of prophets and pastors, expressing the belief that it was senseless to beat patients who were spiritually possessed.

*[I am] disgusted to think of that because we don’t [beat patients] here*, *because the person is suffering and they are already afflicted*, *so how [can] you inflict pain on [such] a person*? *We have never done that and will never do that*.

—Prophetess

Prayer camp staff described using chains on patients with mental illness, and chained patients were observed by the researchers in all but one camp. The staff expressed the perspective that chains served purely the practical purpose of preventing patients from running away or causing harm to themselves or others. Staff indicated that they lacked the sufficient human resources or physical structures to supervise patients with serious mental illnesses and that recovering escaped patients was difficult, given the rural location of the camps. Without security walls and gates around the camp, chains provided a low-cost means of ensuring that patients could not abscond. Chains were generally anchored to cement and were often typically attached to an individual’s leg, around the ankle. At one camp, socks were used to provide padding on the chains; in general, however, most chains were bare against the legs of patients, causing observable bruising and lacerations.

Biomedical providers expressed deep concern about patients being encouraged to fast, as lack of food and water could exacerbate conditions and lead to poorer health outcomes, and they identified chains as one of the most problematic practices of prayer camps. The use of caning and corporal punishment was also identified in interviews with biomedical staff as a substantial cause of concern. To one nurse, the practice compromised human dignity and were seen as primitive in contrast to biomedical care:

*The chains*, *we reject it totally…We also ask [the camps] not to*, *because when you chain [patients]*, *who checks whether there’s abrasion or wound there*? *Nobody checks… [Even if the chain is bandaged w]e still have problem with chaining*. *The chain itself we see as inhuman*. *Because of our training*, *chaining and other kind of treatments are primitive*… *[In t]he modern way*, *we don’t agree with it*.

—Nurse

### Collaboration between faith healing and medicine

Both prayer camps and biomedical care providers expressed enthusiasm, in general terms, at the prospect of partnership. Prayer camp staff frequently expressed respect for medical knowledge and for the role of physicians as healers. A quotation from a prayer camp pastor illustrates a belief that medicine was the living expression of God’s power to heal.

*[We] believe that the knowledge*, *the intellect that God has given man*, *which man is [using in] producing drugs*, *is also a gift from God*. *We don’t have to despise it*, *as God has given somebody the gift of healing*. *When we despise the gift of the drugs that God has provided for somebody to be given during sickness*, *then it seems we are not appreciating what God is doing because the knowledge and the wisdom have been imparted … from God*.

—Pastor

While medical treatment for mental illness was viewed as futile in cases where the underlying cause of the illness was spiritual, prayer camp prophets expressed openness to collaboration. Collaboration was seen as a favorable acknowledgment of the spiritual and biomedical dimensions of health, as exemplified by a quotation from a prayer camp prophetess.

*We should work together*, *thus using the hospital and the prayer camps*, *because God created himself in many ways*. *He’s the creator of the universe*. *There is [a] physical side to everything*, *and there is [a] spiritual side*. *Let those in the prayer camp handle the spiritual aspect*, *[and let] the physicians handle the physical aspect*.

—Prophetess

Additionally, prayer camp staff demonstrated some practices that were supportive of biomedical efforts to improve the health of patients. Certain camps permitted patients to take medication for mental illness, with the caveat that taking medication was concurrent with fasting and prayers. One camp provided financial assistance to patients to purchase prescribed drugs, and encouraged new patients to enroll in Ghana’s National Health Insurance Scheme. Prayer camp staff also indicated that health professionals had been invited in the past to give lectures to congregations on health topics, including nutrition, stroke, and hypertension.

Several prayer camps described informal referral relationships with local hospitals and psychiatric units. Prayer camp prophets stated that they advised patients with obvious physical, medical conditions to go to a hospital for evaluation, prior to the beginning of spiritual healing (some camps noted that they sent particularly aggressive patients to mental health hospitals, in order to sedate and calm the patients before accepting them at the prayer camp). One pastor expressed a belief that physicians were the means through which God exerted healing; for that healing to occur, a patient would need to seek out medical care.

*If you have sickness*, *God will not come heal you*. *You must go to the hospital*. *God is all powerful*, *but he gives that power to others to do his work*. *The doctor is like a small God*.

—Pastor

Nevertheless, prayer camp staff occasionally expressed negative attitudes towards biomedical providers. For instance, one pastor indicated a wariness towards doctors and nurses, expressing concern that some health professionals relied too heavily on secular biomedical knowledge and forsook a reliance on God at their peril.

*Some of the doctors*, *they do not believe in God…To the doctors*, *I say*, *let them fear God*. *They should not depend on books*; *they should depend on God*, *and they will become great and powerful*. *I pity the doctor who does not believe in God*.

—Pastor

Church elders associated with prayer camps expressed concerns that the use of medication inhibited the camp’s practice of fasting and prayers, as sedatives left patients unable to pray or to engage with spiritual healing. Prayer camp staff additionally did not endorse the taking of medications over the long-term, as the need to take medication for mental illness as a chronic condition was perceived as evidence that the spiritual problem had not been resolved. Long-term medication use was described as the *management* of illness, rather than its *healing*, which was believed to be possible only through spiritual means, as exemplified in a quotation from a prayer camp church elder.

*When you go to the hospital*, *and they say you are okay*, *that means every month*, *or every three weeks or so*, *you have to go for medicine*. *Without the medicine for two weeks*, *you will fall back into your sickness*… *That is why you are being controlled*. *It is the medicine which is controlling you*. *Here*, *there is no medicine*, *just spiritual healing*. *If you are healed*, *you are healed forever*.

—Church elder

Despite these distinct views about the causes and preferred treatments for mental illness, prayer camp staff spoke enthusiastically about the possibility of collaborating with biomedical providers, especially those working for regional clinics or for non-governmental organizations. One prophet expressed the hope that psychiatrists would come to his camp to assist with extreme cases of serious mental illnesses. Other prayer camp staff expressed openness to visits by community psychiatric nurses to provide assistance to patients with mental illnesses, and to visits by physicians and nurses to help other patients with somatic diseases and medical conditions.

*You see*, *our sole mission is to see people who are sick get better*. *That is our sole thing*, *to see people who are sick*, *who are in trouble*, *and who have sicknesses*, *being better*. *If we are playing that part and then the medical [staff] are also playing that part for the people to get better*, *why aren’t we happy*? *The result is once a person is healed*, *we are all happy*.

—Pastor

Prayer camp staff emphasized the need for assistance in extending dormitories and other structures, requesting aid in the form of money, cement, aluminum sheets, cleaning supplies, mosquito nets, medicine, and mattresses. Camp staff explicitly called for assistance from non-governmental organizations and from biomedical staff in supporting the general work of the church, expanding or creating new structures, providing medical treatment for patients, or helping in the ancillary services of the church, such as education. As exemplified by a quotation from a prayer camp prophetess, camps expressed a willingness to work with medical NGOs and biomedical staff—citing critically needed resources—to provide services for patients with mental illness, even if cooperation entailed setting aside the use of chains and fasting.

*If the [medical] NGO has a better way to keep [patients] safe*, *then we will use it*. *If they need food for their medicine*, *then we will allow it*. *But we* need *help*. *If an NGO could come and help us care for these people*, *it would be a very blessed thing*. *We need doctors*. *We need nurses*. *We need supplies*.

—Prophetess

Similarly, biomedical providers expressed positive opinions towards the potential for collaboration between prayer camps and biomedical facilities; they conveyed respect for patients’ religious beliefs and accepted that patients could benefit if treatment accommodated those beliefs. A quotation from a community psychiatric nurse exemplified one way that partnership between faith healing and use of medicine may be theologically justified. She said,

*God is the healer*, *but you don’t actually look upon God to descend from Heaven and come and heal you*. *He uses people to heal*, *just as how he uses the prophet to put his hand on you to receive prayers*. *The same way he uses doctors and nurses to also help*… *God is our helping*. *That’s why he is helping us to intervene with our medication*.

—Community psychiatric nurse

However, other biomedical providers were averse to potentially collaborating with prayer camps, citing concerns over practices and the treatment of patients with mental illness. As exemplified in a quotation from a community psychiatric nurse, some biomedical care providers were staunchly opposed to associating with prayer camps.

*[A patient] can’t be [at the prayer camps] all alone…maybe what they would do to the patient, it won’t help them…maybe they will chain them, or give them some other treatment that will not help…Sometimes they will chain them. Even when they need—are in need of something—they will just shout at them. Since they maltreat them, we are not part of them*.

—Community psychiatric nurse

The same nurse suggested that relatives of patients with mental illness could go to prayer camps to pray for their family member at a distance, while the patient received medical treatment in the hospital.

## Discussion

We found that both prayer camp and biomedical staff in Ghana had interest in potential intersectoral partnerships to care for patients with severe mental illnesses. Prayer camp staff were interested in partnership in part because they struggle to provide services for patients with serious mental illnesses. Many of the poor conditions at the camp reflected limited availability of resources, rather than conditions which prayer camp staff considered ideal for their patients (e.g., the use of chains, inadequate shelters). This strengthens the possibility of intersectoral partnerships, and draws attention to key areas where the assistance and involvement of the biomedical sector could be leveraged to generate sizable impact on improving the care of patients with serious mental illnesses.

The biomedical providers and prayer camp staff interviewed broadly agreed on several foundational elements of providing care for patients with mental health needs. First, both parties generally agreed that drug use was often entangled in mental health issues and that reducing drug use would be beneficial. In the short term, this might give rise to joint health educational programs and community outreach partnerships between prayer camps and biomedical care providers that identify the determinants and dangers of drug use and help detect indicators of high risk for mental health issues earlier in people’s lives. More broadly, prayer camp staff could be amenable to visits from community health works to provide lectures to congregations on topics related to health and wellness, as well as to potentially provide care for physical conditions to patients residing at the camp. Prayer camp staff indicated a respect for medical knowledge and saw benefit in the joint acknowledgment of the spiritual and biomedical dimensions of health. This suggests the possibility of collaboration in the medium term for integrating some medication use for mental health conditions under the supervision and administration of biomedical providers, who could visit prayer camps regularly to monitor patients on prescribed treatments. Biomedical care providers could also partner with prayer camp staff to build and strengthen existing referral networks of prayer camp attendees to visit district hospitals and community health centers for outpatient medical check-ups and emergency care, which many prayer camp staff and biomedical care providers mutually saw as beneficial for the benefit of patients. Broader support—such as infrastructural investments in security walls, gates, and suitable living facilities—could abate the use of chains and improve living conditions for patients residing at prayer camps; however, government investments directed to prayer camps will be challenging to justify, particularly within a resource-constrained context, and may not align with other extant budget priorities.

Where less agreement was apparent was in the use of long-term medication as a way of treating mental illness, and in the encouragement of fasting for patients. Collaboration in this area to promote adherence to drug regimens and to mitigate the impact of fasting on the patient’s prognosis would require more extensive discussion and joint learning. Partnership efforts that would attempt to entirely separate spiritual and biomedical perspectives vis-à-vis the care and treatment of patients living with serious mental illness would likely prove fruitless, and may be damaging to future efforts of community engagement: both prayer camp staff and biomedical care providers highlighted that—for many patients and, even, for themselves—the experience of mental illness often occurred through both a biomedical and a spiritual lens.

Our research presents one of the largest studies of prayer camps that treat serious mental illnesses, to date. The limited studies on prayer camps to date have described some of the beliefs and practices of faith healers at these camps [6]. Much of the previous literature has focused on the adverse practices conducted in the camps, including forced fasting, chaining, and caning [15,17], practices which have raised concerns regarding the viability of intersectoral partnerships. Although our findings indicate that these practices are neither universally applied nor universally endorsed at prayer camps, our results were consistent with previous studies [6,33] which found these practices to challenge collaboration from the standpoint of biomedical staff. Furthermore, our findings indicated that prayer camp staff have deeply held beliefs regarding the use of fasting and the need to treat spiritually prior to, or current with, biomedical treatment for medicine to be effective. Long-term biomedical treatment was not recognized as helpful by prayer camp staff, echoing findings regarding similar skepticism among patients with mental illnesses in Ghana [34]. These issues may create challenges for potential intersectoral partnerships. In addition, our informants noted that many Pentecostal and Evangelical Christians in Ghana believe it possible to have voice-hearing experiences and not be mentally ill, which echoes similar findings from Luhrmann, Padmavati, Tharoor, and Osei [35]; within biomedical psychiatry, an emerging body of work studying auditory verbal hallucinations has challenged the notion that they are intrinsically pathologic [36,37], suggesting a perspective that could compliment those of Pentecostal or Evangelical patients.

Our findings should be interpreted in light of some limitations. First, the study chose to focus on prayer camps in Ghana, and results may differ in other settings. Given the limited data on faith healers’ dispositions toward mental illness and biomedical partnerships, it was valuable to explore these issues, even within a limited context, in order to identify recurrent themes and inform future policy directives. Second, as is the case in all qualitative research, our findings are limited by the possibility that respondents may not have answered questions honestly. To promote candor, we shared our discussion guide with prayer camps in advance, and we emphasized that study participants could choose to end the interview at any time, to not be recorded, and to not respond to particular questions. Respondents discussed a range of personal and sensitive subjects, and none chose to skip questions, suggesting some level of comfort with the discussions, although more comprehensive ethnographic data gathered over time would be useful. Last, we were not able to talk with patients or family caregivers, whose perspectives would be important to the issue and should be included in future studies of potential partnerships between faith healers and medical providers. Nevertheless, the exclusion of these constituencies allowed us to develop deeper insights as to the potential for collaboration among providers.

Our findings suggest a number of interventions that could improve the quality of patient life in prayer camps, such as providing adequate shelters for patients, raising gated and secure perimeters to abate the use of chains, and delivering medication to the camps, both for mental illnesses and for general diseases and conditions. These interventions could be accomplished through intersectoral partnerships, but cooperation would require engaging members in frank discussions of concerns, goals, and motivations. These discussions may be facilitated by ongoing efforts to develop a Ministry of Health-sponsored regulatory scheme for prayer camps. By acting in concert, biomedical and faith healers might not only overcome resource limitations but also improve the quality of care for patients with serious mental illnesses. Whether such partnerships will succeed in these goals remains to be seen.

In summary, prayer camp staff and biomedical providers are willing to engage each other in order to enable improved conditions for prayer camps patients, and prayer camp staff identified substantial needs in terms of resources that could potentially be addressed by biomedical providers. Nevertheless, substantial challenges to meaningful partnership were apparent in terms of differences in beliefs about etiology and endorsed treatment practices. Biomedical providers’ interest in engaging with prayer camps seemed borne of an interest in reforming prayer camp conditions, which was not a priority for prayer camp leadership. Accordingly, additional discussion is needed to find the common ground on which the scarce resources for mental health care in Ghana can collaborate most effectively.

## Supporting Information

S1 File Discussion guide. This file contains the discussion guide used in interviews with biomedical staff and prayer camp staff as part of the data collection for this study.
(PDF)
