# Supplementary material for: Prayer Camps and Biomedical Care in Ghana: Is Collaboration in Mental Health Care Possible?
Source: PLoS One. 2016 Sep 12;11(9):e0162305. doi: 10.1371/journal.pone.0162305 (PMC5019394; doi:10.1371/journal.pone.0162305)
Supplement: S1 File — This file contains the discussion guide used in interviews with biomedical staff and prayer camp staff as part of the data collection for this study. (PDF) [file pone.0162305.s001.pdf]

Questions for Biomedical Staff:

- Good morning. I would like to begin by asking you a few questions regarding your work at [Pantang/Ankaful/Accra]:
  - What is it that you do at the hospital? (What is your position?)
  - What led you to become a _____ (doctor, nurse, etc.) at [Pantang/Ankaful/Accra]?
  - Without identifying any individuals, could you describe the types of cases that you see in the inpatient and outpatient wards?
  - What does the term "mental illness" mean to you?
  - What do you think causes mental illness?
- I would now like to ask you a few questions regarding your religious beliefs.
  - Are you religious?
  - Could you tell me a little about your religious belief?
    - How—if at all—do your religious beliefs influence or inform your work as a _________ (doctor, nurse, etc.)?
- I would now like to ask a few questions regarding Ghana's prayer camps. Are you familiar with prayer camps that treat mental illness in Ghana?
  - Without identifying individuals, have you had any patients with mental illness that came from prayer camps?
    - Have you ever referred a patient to a prayer camp?
  - Have you, in your capacity as a _________ (doctor, nurse, etc.), visited a prayer camp? If so, what was it like for you?
  - How, to the best of your knowledge, do the prayer camps treat people with serious mental illness?
  - What do you think of faith healing for mental illness?
  - (If the participant has indicated a negative view of prayer camps) Could the situation in prayer camps be improved? How so?
- I would now like to ask a few questions regarding traditional healers.
  - Without identifying individuals, have you had patients referred from traditional healers?
    - Have you ever referred a patient to a traditional healer?
  - Have you, in your capacity as a _________ (doctor, nurse, etc.), ever worked with a traditional healer? If so, what was the experience like for you?
  - How, to the best of your knowledge, do traditional healers treat people with serious mental illness?
  - What do you think of traditional healers and traditional medicine?
  - (If the participant has indicated a negative view of traditional healers) Could the situation with traditional healers be improved? How so?
- Is there anything else you'd like to discuss, or any questions you have for me?

Questions for Prayer Camp Staff:

- Good morning. I would like to begin by asking you a few questions regarding the camp.
    - What is the camp's name?
    - What is the camp's affiliation? Does it have a particular denomination?
    - Who is or are the leaders of the camp?
    - For how long has the camp been in operation?
    - How many people are part of the camp's full-time staff?
    - How many people visit the camp daily/weekly? Is there a congregation affiliated with the camp?
    - How many people reside at the camp as patients?
    - Do any community psychiatric nurses or other mental health providers visit the camp?
    - Do any doctors or nurses visit the camp?
    - How many buildings are part of the camp compound? How many are used by people staying at the prayer camp?
    - What types of conditions do people coming and residing at the prayer camp have?
    - What is the average gender distribution of patients residing at the camp? Age distribution?
    - Are patients residing at the camp asked to pay money for their stay? If so, how much? If not, how are the costs related to their care paid for?
    - Does the camp refer patients to hospital, other prayer camps, or any other facility?
- I would now like to talk about your work at the camp
    - What is it that you do at the camp? (What is your position?)
    - What led you to become a _____ (pastor, caretaker, prophet) at the camp?
    - Without identifying any individuals, could you describe the types of cases that you see at the camp during an average day?
    - What does the term "mental illness" mean to you?
    - What do you think causes mental illness?
- I would now like to ask you a few questions regarding your religious beliefs.
    - Are you religious?
    - Could you tell me a little about your religious belief?
        - How—if at all—do your religious beliefs influence or inform your work as a _________ (pastor, caretaker, prophet, etc.)?
- I would now like to ask a few questions regarding what it is that you do here. Can you tell me a little about treatment at the prayer camp?
    - How may an average person coming to the camp with mental illness be treated at the camp?
    - Without identifying individuals, have you had any patients with mental illness that came from hospital? From traditional healers? From other prayer camps?
        - Have you ever referred a patient to a hospital, doctor, traditional healer, prayer camp?

- At some camps, patients with mental illness are chained. Could you tell me if this is something that happens at [name of prayer camp]?
  - If so, could you tell me more about why chains are used to restrain patients with mental illness at the camp? If not, could you tell me why you do not use them?
- At some camps, patients with mental illness fast. Could you tell me if fasting is encouraged at [name of prayer camp]?
  - If so, could you tell me more about your beliefs regarding the use of fasting for patients with mental illness? Are there patients that you do not encourage to fast? Are others allowed to fast on the behalf of patients?
- At some camps, patients with mental illness are caned or physically struck. Could you tell me if this occurs at [name of prayer camp]?
  - Could you tell me what caning means for you? If patients are caned, could you tell me why?
- What do you think of medical healing for mental illness? Faith healing for mental illness? Traditional healing for mental illness?
- What do you think about collaboration with medical healers to provide care for patients at the prayer camp? What would you be interested in? What would you be concerned about?

- Is there anything else you'd like to discuss, or any questions you have for me?
